# Supplementary material for: An alternative pathway of enteric PEDV dissemination from nasal cavity to intestinal mucosa in swine
Source: Nat Commun. 2018 Sep 19;9:3811. doi: 10.1038/s41467-018-06056-w (PMC6145876; doi:10.1038/s41467-018-06056-w)
Supplement: Supplementary file 1 — Supplementary Information [file 41467_2018_6056_MOESM1_ESM.docx]

**Supplementary Information**

**An alternative pathway of enteric PEDV dissemination from nasal cavity to intestinal mucosa in swine**

Li, et al.


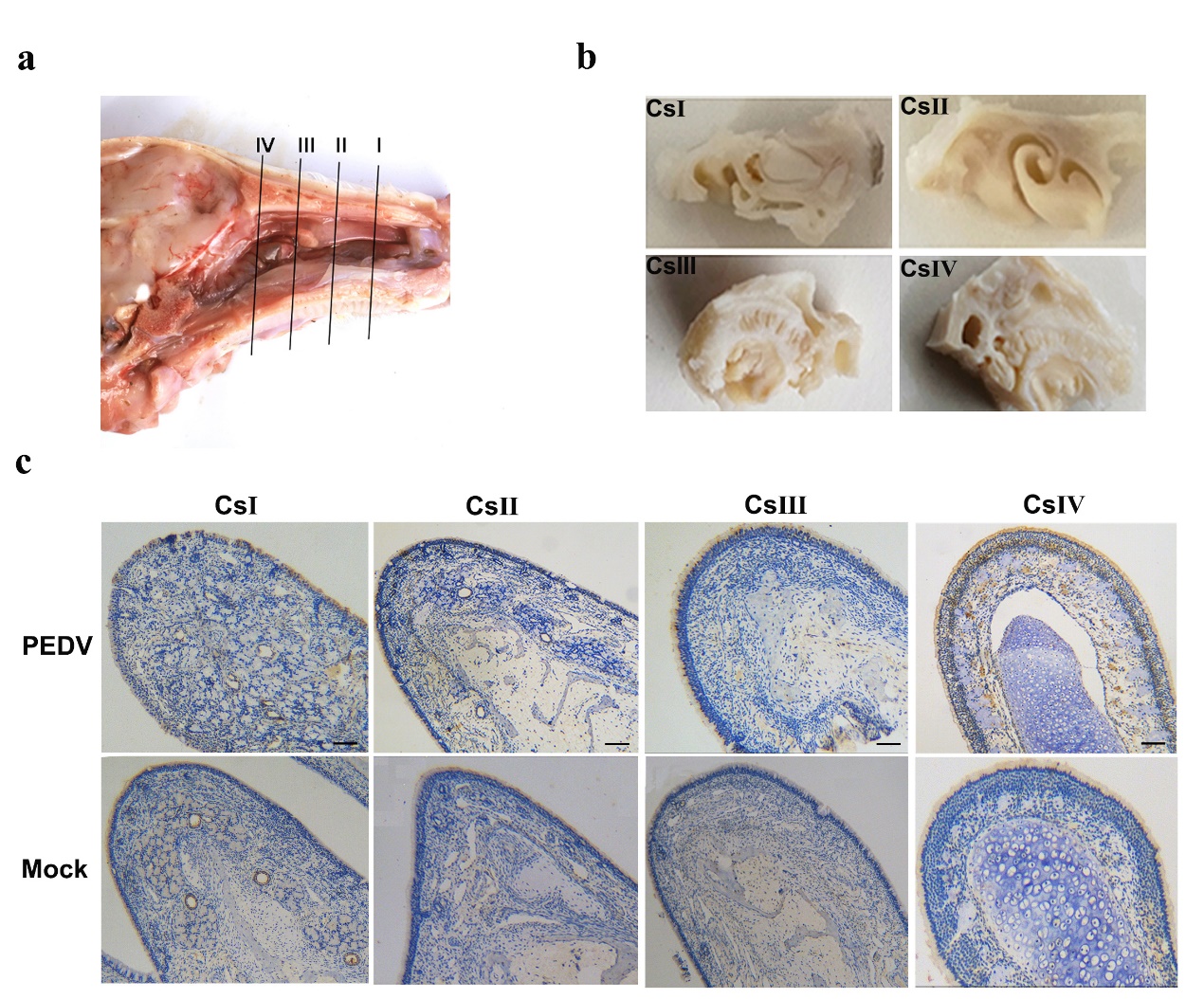
**Supplementary Figure 1. PEDV positive epithelial cells were observed in four cross-sections of middle nasal concha.** **a** Diagrams of the four selected cross-sections (I±IV) of the piglet’s nasal cavity. Four cross-sectional blocks were choosed according to structure of pig nasal cavity: the vestibular region (I), regio respiratoria (II, III), and regio olfactoria (IV). **b** The anatomical structure of the four cross-sections. **c** Distribution pattern of PEDV in middle nasal concha of piglets’ nasal cavity at low magnifications. Bars: 200 μm.

**
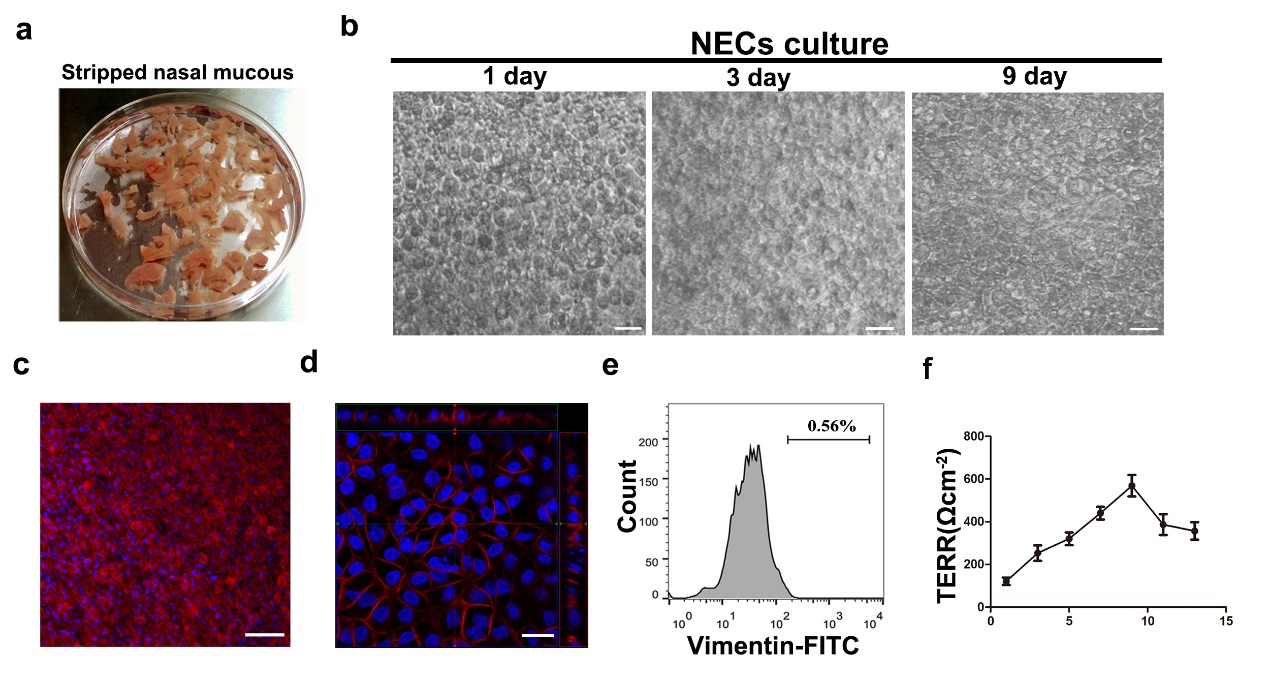
**

**Supplementary Figure 2. Evaluation of NECM establishment.** **a** Stripped nasal mucous. **b** Air liquid interface was establishing and observing on 1, 3, 9 days after seeding. **c** Epithelial cells in NECM was staining and observed by CLSM. Keratin 18 was shown in red. Cell nuclei were stained with DAPI (blue). **d** The cross-sectional images obtained by CLSM showed the distribution of PEDV receptor APN in NECM. APN was shown in red. Cell nuclei were stained with DAPI (blue). **e** Dissociated cells from NECM detected by the fibroblast cell marker vimentin. Bars: 20 μm. **f** The transepithelial electrical resistance (TEER) of NECM after seeding. Data express mean ± SD from three samples.

**
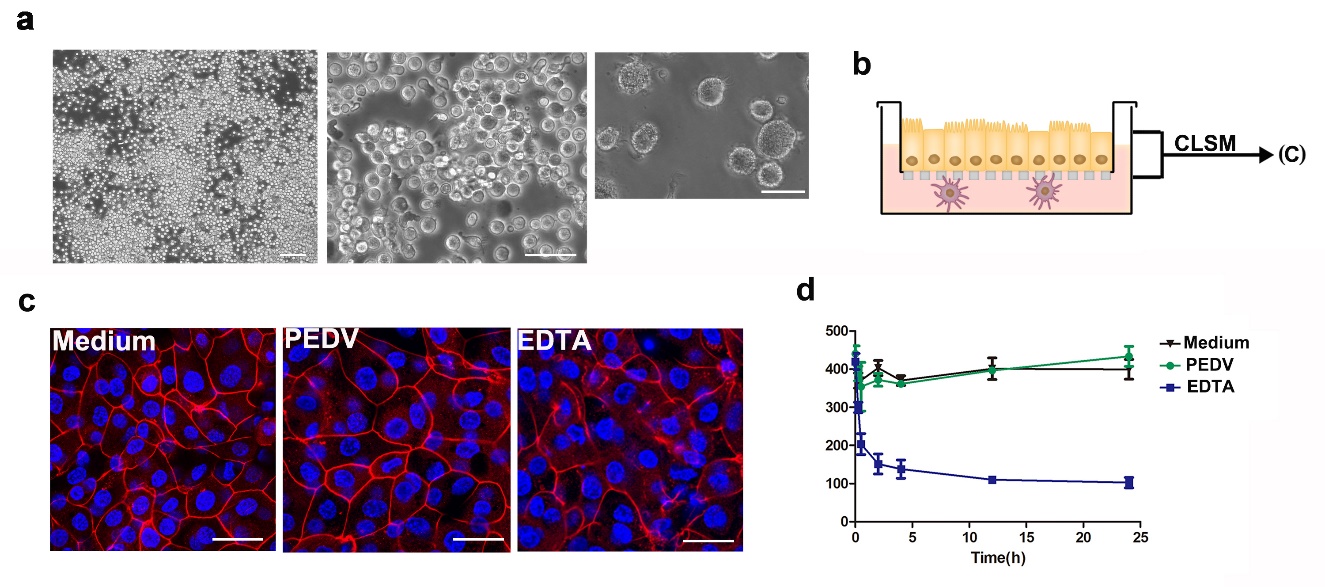
Supplementary Figure 3. Barrier integrity of NMEs monolayer after co-culturing with DCs and PEDV. a** Morphology of clusters DCs cells (left), immature (Middle) and mature DCs (right) were analyzed via microscopy after 6 days of culture. **b**, **d**  After DCs seeding, the co-cultured model was incubated with medium, PEDV, and EDTA (12.5mM). **c** Tight junction protein ZO-1 (Red) was detected by Immunofluorescence. **d** TEER was measured using a voltohmmeter at different times. Bars: 20 μm. Data express mean ± SD from three samples.

**
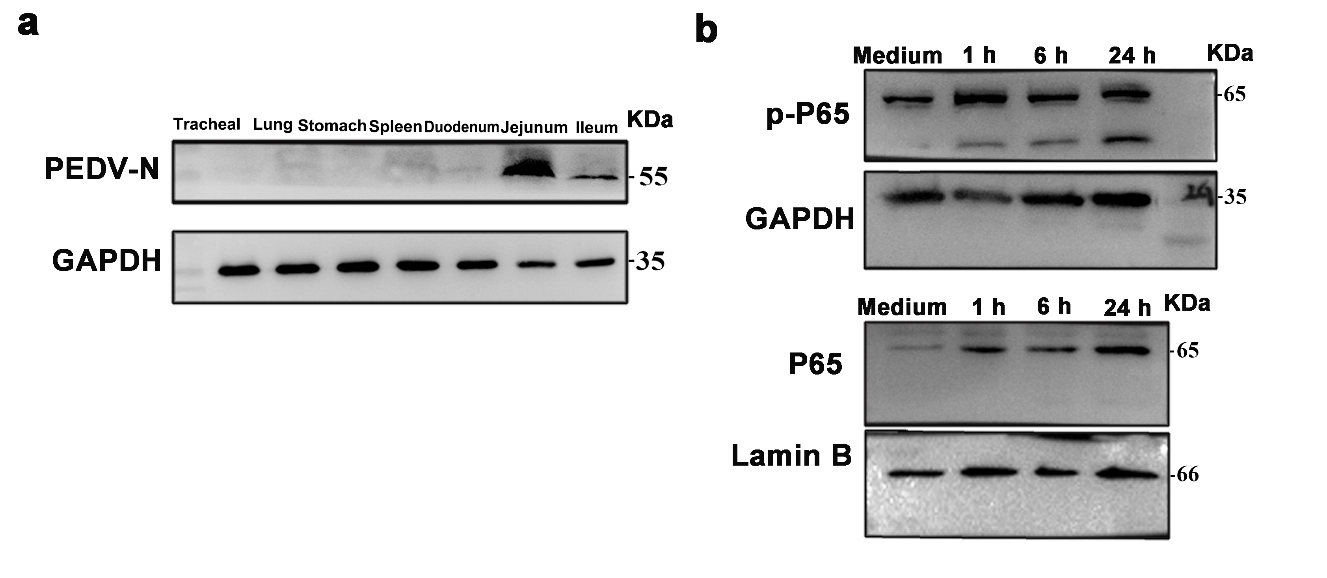
**

**Supplementary Figure 4.** Full western blots. Blots for Figures 1 d(a), and 5e (b).

**
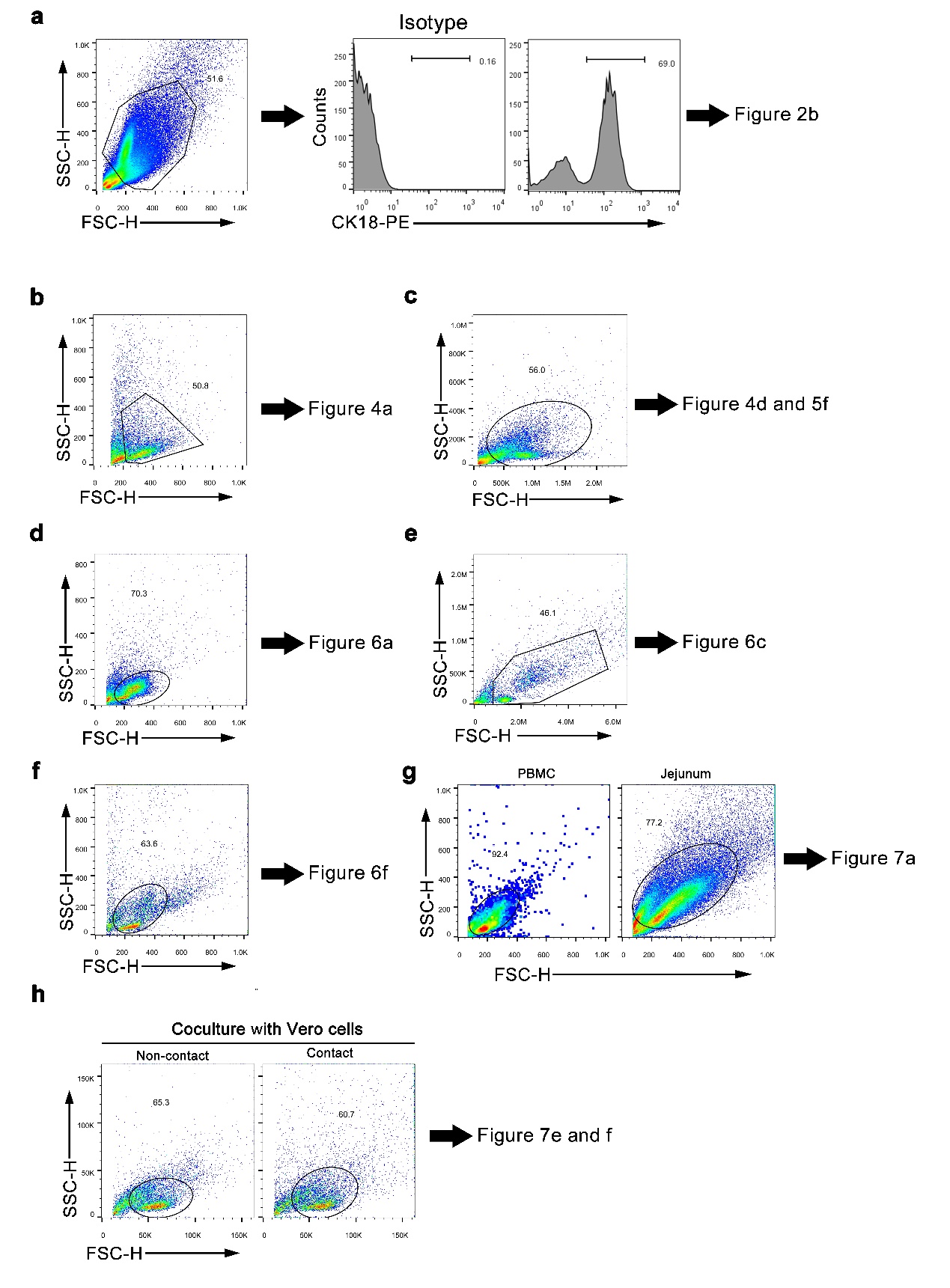
Supplementary Figure 5. Schematic gating strategy of flow cytometry.** Gating strategies used for cell sorting. **a** Relates to Fig. 2b. **b** Relates to Fig. 4a. **c** Relates to Fig. 4d and 5f. **d** Relates to Fig. 6a. **e** Relates to Fig. 6c. **f** Relates to Fig. fc. **g** Relates to Fig. 7a. **h** Relates to Fig. 7e.

**Supplementary Table 1 Primer sequences used for qRT-PCR.**

| Target genes | Primer sense (5’-3’) | Primer antisense (5’-3’) |
| --- | --- | --- |
| PEDV-M | ATGCATGGGCTAGCTTCCAG | GTAGTGAGAAGCGCGTCAGT |
| CCL4 | GCAAGACCATGAAGCTCTGC | AAGCTTCCGCACGGTGTATG |
| CXCL2 | CCGTGCAAGGAATTCACCTC | TGCGGGGTTGAGACAAACTT |
| CCL25 | GCCTACCACAGCCACATTAAG | GCTTCCCGCACACCATCTT |
| CCL20 | GGTGCTGCTGCTCTACCTCT | GCTGTGTGAAGCCCATGATA |
| CCL5 | ATCAGCCTCCCCATATGCCT | CCGCACCCATTTCTTCTCTG |
| CCL3L1 | CTTCCTCGCAAATTCGTAGC | GCATTCAGCTCCAGGTCAG |
| CCL22 | TTACAGCTGCTCCCTGACAC | TGGATTGGAAGGGTAAGAGG |
| CCL2 | GCGGCTGATGAGCTACAGAAG | CCGCGATGGTCTTGAAGATC |
| GAPDH | TCATCATCTCTGCCCCTTCT | GTCATGAGTCCCTCCACGAT |
